# Can existing unrelated vaccines boost a COVID-19 vaccine prime?

**DOI:** 10.1016/j.eclinm.2021.100758

**Published:** 2021-02-11

**Authors:** Nathaniel Hupert, Daniela Marín-Hernández, Douglas F. Nixon

**Affiliations:** a Departments of Population Health Sciences and of Medicine, Weill Cornell Medicine, and Cornell Institute for Disease and Disaster Preparedness, New York, NY, USA; b Division of Infectious Diseases, Department of Medicine, Weill Cornell Medicine, New York, NY, USA

Commentary

The development of new specific vaccines against COVID-19 has occurred as the US program name promised, at “warp speed,” and emergency use authorization has led to vaccine rollout around the world. However, the emergence of new more contagious variants combined with limited vaccine supply has created increased urgency to vaccinate as many people as possible, spurring an international debate about the wisdom of abandoning or delaying currently mandated second dose “boosts” [1,2]. Vaccination is thought to work by generating short-lived antigen-specific cells, some of which then become memory cells with capacity for accelerated expansion upon re-exposure to the same vaccine antigen. Thus, most vaccinations require one or more “boosts” to reach a level of memory cells needed to provide protection against infection. Vaccination schedules are designed around specific trials and regimens, and the currently authorized COVID-19 vaccines have been developed with a booster dose scheduled a certain time after the initial prime.

Economic and logistical considerations have motivated studies to assess one-dose compared to two-dose vaccines, and at least one ‘one-dose’ COVID-19 vaccine is under development. The current push for single dose vaccination has been driven by fear of the rapidly expanding pandemic and with the guess that the immunogenicity of a single dose vaccine and resulting immunity might provide an adequate level of efficacy. As data is being collected on the potential effectiveness of using only the first half of authorized two-dose vaccines, the global urgency to ‘do something now’ has taken us into realm of the immunological unknown: multiple countries already have initiated single-dose regimens (by design in the UK, possibly by default in the US due to newly-discovered gaps in second-dose stockpiles). The classic paradigm for vaccine efficacy is the induction of long-lived antigen-specific adaptive immune responses, in this case through use of a COVID-specific second vaccination dose. But is there a middle way between that approach—which may be logistically infeasible for the foreseeable future—and nothing?

We believe that the concept of heterologous immunity provides a scientifically sound third option. Current licensed vaccines could potentially have non-specific beneficial effects on SARS-CoV-2 infection or COVID-19 disease course through a variety of mechanisms including boosting of trained immunity [3] or increased natural killer cell function and enhanced production of interferon gamma [4]. There is a growing body of evidence which suggests that unrelated vaccines can diminish COVID-19 disease [5]. Much attention has focused on the use of the Bacillus Calmette-Guérin (BCG) vaccine, which provides cross-protection against related and unrelated pathogens through boosting natural and innate immunity by epigenetic reprogramming or “training” of innate immune cells [6]. The BCG vaccine can also independently alter adaptive immune responses to unrelated pathogens and antigens through cross-reactivity and bystander activation. During bystander activation, adaptive immune cells with weak receptor binding of the secondary antigen or no co-stimulation are activated due to signals from an ongoing immune response. These signals may include cytokines which activate T-cells and secondarily increase numbers of unrelated plasma cells.

While prospective trials of BCG vaccination to prevent COVID-19 disease are ongoing, an interesting unrelated study initiated in the pre-COVID era that tested whether BCG vaccination in the elderly protected against infections, has published interim results. This phase 3 randomized clinical trial, called ‘ACTIVATE’, showed protection against respiratory tract infections of probable viral origin, [7] leading to a reasonable chance, albeit still speculative, that BCG vaccination may also reduce COVID-19 morbidity, [8] although that was not formally tested in the ACTIVATE trial. Other vaccines, such as OPV or IAV are also potential agents to protect against COVID-19, [5] and the use of additional non-specific vaccines are being explored [9]. It may turn out that the type of non-specific vaccine used for this purpose is less important than which adjuvant (typically an inert ingredient designed to boost immune reaction to the shot) it contains. Adjuvants have been used in most vaccines and they come in different forms with different modes of action [10]. They are not normally used outside of vaccine formulation, although in oncology and immunotherapy treatments, novel pathogen-associated molecular pattern (PAMP)-adjuvants are being trialed as immunogenetic augmenters.

We propose that non-specific vaccination could be undertaken to immunologically boost the response to COVID-specific vaccines. Put plainly, this could be as anodyne as recommending that populations worldwide “catch up” on recommended non-COVID-19 vaccines one month after their COVID jab. Such “heterologous vaccine boosting” (HVB) should only be considered if an indicated second COVID-19 vaccine specific boost is not available. Had we the luxury of time, careful pre-clinical studies to investigate the viability and effect of HVB would be in order, but animal model studies could quickly test this hypothesis. Given the urgency of the moment, we believe that, even absent such studies, a non-specific vaccine boost for a COVID-19 prime would be preferable to no boost, and using existing vaccines such as IAV, OPV or BCG which provide benefit in their own right, could be implemented at “warp speed”. HVB with existing vaccine stocks of IAV, OPV or BCG vaccines are ready now, and could circumvent delays in specific vaccine production.

## Declaration of Competing Interest

Authors declare no competing interests.
